# Recurrent bacteremia with *Enterococcus faecalis*, the clinical findings predicting endocarditis, and genomic characterization of the isolates: a retrospective cohort study

**DOI:** 10.1007/s10096-023-04636-3

**Published:** 2023-07-08

**Authors:** Chaitanya Tellapragada, Helena Östlund, Christian Giske, Magnus Rasmussen, Andreas Berge

**Affiliations:** 1 Division of Clinical Microbiology, Department of Laboratory Medicine, Karolinska Institutet, Stockholm, Sweden; 2 Unit of Infectious Diseases, Department of Medicine, Solna, Karolinska Institutet, Stockholm, Sweden; 3 Department of Clinical Microbiology, Karolinska University Hospital, Stockholm, Sweden; 4 Division of Infection Medicine, Department of Clinical Sciences Lund, Lund University, Lund, Sweden; 5 Division for Infectious Diseases, Skåne University Hospital, Lund, Sweden; 6 Department of Infectious Diseases, Karolinska University Hospital, Stockholm, Sweden

**Keywords:** *Enterococcus faecalis*, Bacteremia, Endocarditis, Recurrent infection, Whole genome sequencing, Sequence type

## Abstract

Multiple episodes of *Enterococcus faecalis* bacteremia (EfsB) may indicate a relapse and be due to an undiagnosed infective endocarditis (IE). The aims were to study the clinical presentation of patients with EfsB with focus on the risk of recurrent infection and IE, identify potential improvements of the management, and to investigate whether *E. faecalis* isolates from different episodes in the same patient were identical. In a retrospective study, a cohort of patients with monomicrobial (M) EfsB episodes was analyzed. Clinical data from medical records were collected. Furthermore, blood culture isolates from patients with multiple episodes were subjected to whole genome sequencing and multilocus sequence typing. In 666 episodes of MEfsB, 69 patients with IE and 43 with recurrent infections were found. Patients without IE, but with a following episode diagnosed as IE, were compared to those without a following episode. Variables significantly correlated with IE were long duration of symptoms, growth in all blood cultures, unknown origin of infection, heart murmur, and predisposition for IE. Transesophageal echocardiography, all negative, was done in 4 out of 11 episodes during the first episodes, later diagnosed with IE. In 28 of 31 patients with two or more EfsB episodes, isolates with identical sequence type were found. Episodes of EfsB in patients later diagnosed with IE showed features of IE already during the first episodes, were not adequately evaluated, are due to identical isolates, and most likely represent true relapses. Risk factor analysis should guide the use of echocardiography.

**Supplementary Information:**

The online version contains supplementary material available at 10.1007/s10096-023-04636-3.

## Introduction

*Enterococcus faecalis* (Efs) is the species that cause the most *Enterococcus* clinical infections [1]. Efs can cause urinary tract infections (UTIs), cholangitis, cholecystitis, diverticulitis, and other abdominal infections along with nosocomial infections as catheter-related infections (CRIs) [1]. These and other infections can constitute the origin of and an entry point for the bacterium into the blood stream, resulting in bacteremia. Bacteremia with Efs (EfsB) is a common and important situation in many clinical contexts [2–4], and the risk of bacterial hematogenous spread to joints, bones, and, most importantly, the heart has to be evaluated [5–7].

The most important complication to bacteremia with *Enterococcus* is infective endocarditis (IE), with considerable mortality [4], and Efs is the dominating *Enterococcus* causing IE [4, 5, 8]. IE is primarily diagnosed by positive blood culture (BC) and detection of changes on cardiac structures, indicating IE [9]. To detect these changes, transesophageal echocardiography (TEE) is by far more sensitive than transthoracic echocardiography (TTE) (92% and 37–43%) [5, 10] but also positron emission tomography–computed tomography (PET-CT) and cardiac multi-slice CT can be used [11].

One of the risk factors for Efs IE is monomicrobial (M) bacteremia [2, 6, 8]. Other risk factors are long duration of symptoms (D), embolization (E), growth in all or the majority of the BC bottles (N), unknown origin of infection (O), predisposing cardiac condition (V), auscultation of heart murmur (A), community acquisition, and immunosuppression [2, 4, 6, 8, 10, 12, 13]. “The NOVA score”, a risk factor acronym, can be used to evaluate the risk of IE [5]. The NOVA score has a high sensitivity but is hampered by a low specificity and high number needed to screen to find cases of IE [5–7]. To improve the performance of NOVA, the variables independently correlated to IE in episodes of MEfsB were identified, and through adding long duration of symptoms (D) and embolization (E), a score system called the DENOVA score was developed [7]. A history of a previous episode of EfsB correlated to IE in a following episode of MEfsB but was not independently correlated in the multivariate analysis [7] and was thus not included in the DENOVA score.

Recurrent episodes of EfsB have been noted in many studies, mainly as relapses after IE, but the variables correlated to relapse as an isolated outcome in patients with bacteremia have not been specifically addressed, except for the description of insufficient evaluation. However, the treatment time of IE patients correlate to relapse [2, 8, 10, 14–17].

A previous study of recurrent polymicrobial and MEfsB episodes using pulsed-field gel electrophoresis showed that 8/27 of the patients were infected with identical or similar isolates in both episodes [18]. IE, UTIs, CRIs, and the intestines were the foci during the relapses. Dahl et al. [10] found 20 recurrent infections after 344 episodes of EfsB, 7 after IE (8%) and 13 after non-IE episodes (5%), but the diagnoses of the following episode were not described. In that study, all patients were subjected to echocardiography and 76% to TEE.

The Efs isolates can be divided into different sequence types (STs) either by sequencing multiple PCR products or by whole genome sequencing (WGS). Multilocus sequence typing (MLST) is a robust typing method that is widely used to study the genetic relatedness of bacterial isolates based on sequencing data of seven housekeeping genes [19].

In this study of multiple episodes of EfsB, in a post hoc analysis of data from the DENOVA study [7], we have described the clinical characteristics of the different episodes, with focus on risk factors for IE and other variables correlated to IE. Furthermore, we hypothesized that consecutive isolates of Efs in patients diagnosed with IE in the last episodes would most likely be of the same ST, thus suggesting that these isolates were genetically identical. Identical isolates in recurrent infections should indicate a relapse of an inadequately treated infection during the preceding episode, possibly an undiagnosed IE. To investigate this hypothesis, we performed WGS of coupled isolates taken from different episodes in the same patient, followed by in silico MLST analysis.

## Materials and methods

The study cohort was a compilation of the calibration and validation cohorts from Berge et al. [7], a population-based cohort of patients from the Skåne region, Sweden, and a cohort from a tertiary referral center, Karolinska University Hospital, Stockholm, Sweden. The cohort was defined by all patients with MEfsB consecutively collected from the databases of the Clinical Microbiology Laboratory in Region Skåne and from Karolinska University Laboratory, Karolinska University Hospital, Stockholm, Sweden, with MEfsB from January 2012 to December 2016. Clinical and microbiological data from 1 year before to 1 year after the bacteremia were collected [7]. Ethical approval was obtained from the Ethics Committee Review Board in Stockholm (Dnr 2015/1184-31) and from the Ethics Committee of Lund University (2013/31), both later substituted by the Swedish Ethical Review Authority after a reorganization.

### Definitions

An episode of EfsB was defined by the growth of Efs in at least one BC bottle and a clinical situation where the patient seeks medical attention for any symptoms compatible with EfsB. Multiple BCs taken on different days were considered to belong to the same episode if they were taken during the same clinical situation. An episode was delimited by at least 7 days of effective antibiotic treatment and clinical improvement or, in case of the absence of improvement and lack of positive BC, 30 days. Recurrent infection or recurrence was defined as an EfsB found after the delimitations of an episode and a relapse as a subgroup of recurrent infections, defined as a recurrent B due to the same isolate.

A MEfsB was defined as an episode with the growth of Efs in at least one BC bottle without other bacteria isolated in any of the BCs during the episode, except for the growth of coagulase-negative staphylococci in no more than one of the BC bottles. Polymicrobial (P) EfsB was defined as any episode with the growth of Efs in BC together with another organism and not fulfilling the criteria for MEfsB.

Other definitions, for example of the origin of infections and focal infections used, were as in Berge et al. [7] including its supplementary material. The NOVA and DENOVA scores were calculated as described [5–7]. To define definite, possible, and rejected IE, the ECS criteria (derived from the modified Duke criteria [9]) published during the study period were used [11].

### Microbiology

All retrievable isolates of Efs from BCs stored in the laboratories’ isolate collections were cultured on blood agar plates. Identity of all the isolates as Efs was confirmed using MALDI-TOF MS (Bruker Diagnostics). Genomic DNA was extracted [20] from freshly subcultured colonies of Efs using EZ1 Advanced XL system (Qiagen, Inc., Hilden, Germany). The extracted DNA was quantified using Qubit dsDNA assay kit (Thermo Fisher, Waltham, USA). Sequencing libraries were prepared using Nextera XT kit (Illumina, San Diego, CA, USA) as per the manufacturer’s instructions. Whole genome sequencing was performed on HiSeq 2500 system (Illumina) at the Science for Life Laboratory, Stockholm, Sweden. Quality of the WGS data was verified using FastQC v0.11.8, and assembly of the trimmed reads was performed using SPAdes v3.13.1. MLST of the isolates was performed using MLST v.2.0.4 at https://cge.cbs.dtu.dk/services/MLST/. Whole genome assemblies of isolates with novel allelic profiles were submitted to the PubMLST database. Genome sequences of the isolates with new MLST profiles assigned from this study are available on the Efs PubMLST database (https://pubmlst.org/organisms/enterococcus-faecalis).

### Clinical data

Clinical data were collected by the study of medical records. A detailed description of the clinical data collected is given in Berge et al. [7] and its supplementary material. Data on the results of evaluation with PET-CT and cardiac CT were collected.

### Statistical analysis

The analysis of the collected data was calculated in the statistical program Stata, version 15.1 (StataCorp, College Station, TX, USA). The odds ratios and their confidence intervals were calculated when applicable. The chi-square test was used when the prerequisites were met, and Fisher’s exact test was used otherwise. Differences between continuous variables were analyzed with Wilcoxon’s rank sum test. Values are presented as proportions with percentage or medians with interquartile ranges. A *p* value of < 0.05 was considered to be an indication of a significant difference.

## Results

### Description of the cohort

The cohort consisted altogether of 666 episodes of MEfsB in 616 patients. Of these episodes, 69 were diagnosed as IE (10%). Another 168 cases fulfilled the clinical criteria for possible IE, and in 51 of these, the IE diagnosis was rejected based on another diagnosis, resulting in 117 episodes of possible IE remaining.

During the study period, there were a total of 566 patients with only one episode of MEfsB, 43 patients with at least two episodes of MEfsB, and seven patients with one MEfsB and one PEfsB (Table 1, row numbers 1–5). Among these 43 patients, 36 patients had two episodes of MEfsB, seven patients had three episodes, and no patient had more than three episodes (Table 1). A description of the patients with polymicrobial and monomicrobial infection episodes has been given in the Supplementary (S) Material.

**Table 1:** Schematic representation of the cohort: categories of patients with different combinations of one, two, or three MEfsB episodes and, if present, a preceding or following PEfsB

	Preceding PEfsB	Preceding MEfsB 1	Preceding MEfsB 2	Last episode MEfsB	Following PEfsB	Number of patients
1	X			X		5
2	X	X		X		2
3		X		X		34
4		X	X	X		7
5				X	X	2
6				X		566

**Fig. 1:**
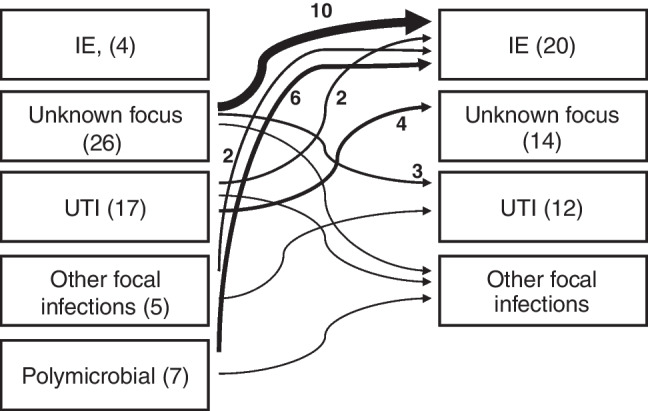
Schematic representation of the diagnoses of the episodes. Patients with different diagnoses in different episodes are indicated by arrows. The first column shows the diagnoses of all episodes preceding an EfsB including the PEfsB. The second column shows the diagnoses during the last episode in each patient. A single patient can have multiple preceding episodes and an episode can have more than one diagnosis, but why arithmetics are not fully applicable. The arrows indicate the different diagnoses in the episodes including the PEfsB. The width of the arrow indicates the number of patients, and numbers > 1 are shown adjacent to the arrow. Patients with the same diagnose in all episodes are not shown with arrows. The number of episodes in parenthesis in each box

All 57 MEfsB episodes preceded by MEfsB or PEfsB (Table 1, rows 1–4, columns 2–4) were compared to 609 episodes in the patients without a previous episode (S Fig. 1). Significant differences in univariable analysis were found in long duration of symptoms, predisposition of IE, heart murmur, embolization, growth in all or the majority of two or more BCs, unknown origin of infection, and healthcare-associated acquisition (S Table 1). A positive DENOVA score was significantly more common. IE was diagnosed in 20 of the 57 episodes (35%), and IE was more common (*p* < 0.001) (S Table 1).

The diagnoses from the 109 episodes in patients with two or more PEfsB (nine episodes) or MEfsB (100 episodes) episodes (Table 1 and S Table 1) are presented in Fig. 1. Four patients had two episodes of IE. Out of 26 patients with unknown origin of infection during the first episode, 10 were diagnosed with IE during the final episode (Fig. 1). Most patients with UTI had UTI during all episodes. From seven episodes of polymicrobial infection followed by a MEfsB, six were IE, and one was diagnosed with spondylodiscitis, during the last episode.

The duration of time between the two episodes varied from 15 to 351 days with a median time to a new episode of 56 days (IQR 31 to 98) (S Fig. 2). The median time was not significantly different whether diagnosed with IE during the last episode or not (69 days (IQR 54 to 116) and 48 days, respectively (IQR 38 to 97), *p* value 0.18).

### Can a recurrent infection be predicted during the first episode?

Patients with a MEfsB with a later MEfsB were compared to patients without a following MEfsB. Episodes in patients not surviving 30 days after the EfsB were excluded, due to not being at risk of a following Efs infection and therefore not possible to classify, and all episodes preceded by an EfsB were excluded (S Fig. 1). Univariable analysis was used to identify clinical characteristics associated with a later recurrent EfsB (Table 2). The analysis identified duration of symptoms of more than 6 days, predisposition for IE, heart murmur, growth in all or the majority of BCs, and unknown origin of the infection to be significantly associated to recurrent infection (Table 2). Furthermore, positive DENOVA score and possible IE were also associated with recurrence. However, treatment time was not associated to recurrent infection (Table 2).

**Table 2:** Univariable analysis of clinical characteristics, management, and diagnosis of episodes followed by a recurrent episode compared to episodes without

Characteristics	Episodes followed by new episodes (*n* = 41)	Unique episodes (*n* = 485)	Odds ratio (95% CI)	*p* value
Acquisition				
Community-acquired	12 (29)	128 (26)	1.2 (0.6–2.3)	0.69
Healthcare-associated	21 (51)	220 (45)	1.3 (0.7–2.4)	0.47
Nosocomial	8 (20)	137 (28)	0.6 (0.3–1.4)	0.23
Duration of symptoms ≥ 7 days	13 (32)	64 (13)	3.0 (1.5–6.2)	*0.001*
CIED	3 (7)	53 (11)	0.6 (0.2–2.2)	0.47
Predisposition	15 (37)	96 (20)	2.3 (1.2–4.6)	*0.011*
Predisposing cardiac condition	14 (34)	87 (18)	2.4 (1.2–4.7)	*0.011*
Prosthetic valve	9 (22)	47 (10)	2.6 (1.2–5.8)	*0.030*
Native valve disease	10 (24)	49 (10)	2.9 (1.3–6.2)	*0.016*
Previous IE	2 (5)	16 (3)	1.5 (0.3–6.7)	0.64
Intravenous drug user	2 (5)	11 (2)	2.2 (0.5–10.3)	0.27
Heart murmur	15 (37)	74 (15)	3.2 (1.6–6.3)	*< 0.001*
Fever ≥ 38°	32 (78)	411 (85)	0.64 (0.3–1.4)	0.26
Embolization	1 (2)	6 (1)	2 (0.2–17)	0.44
Number of positive cultures ≥ 2	20 (49)	114 (24)	3.1 (1.6–5.9)	*< 0.001*
Unknown origin of infection	23 (56)	151 (31)	2.8 (1.5–5.4)	*0.002*
Origin of infection	18 (44)	322 (66)	0.40 (0.2–0.8)	0.004
Gastrointestinal	1 (2)	72 (15)	0.1 (0.02–1.1)	0.03
Urinary tract	14 (34)	206 (42)	0.7 (0.4–1.4)	0.30
CRI	1 (2)	22 (5)	0.5 (0.1–4)	1.0
Wound	0 (0)	16 (3)	n/a	0.63
Other foci	0 (0)	14 (3)	n/a	0.27
Joint infection	0 (0)	3 (1)	n/a	1.0
Spondylodiscitis	0 (0)	9 (2)	n/a	1.0
Spleen abscess	0 (0)	1 (0.2)	n/a	1.0
DENOVA score positive	20 (49)	93 (19)	4.0 (2.1–7.7)	*< 0.001*
Management				
TTE performed	16 (39)	144 (30)	1.5 (0.8–2.9)	*0.021*
TEE performed	11 (27)	96 (20)	1.5 (0.7–3.1)	*0.031*
TTE or TEE performed	19 (46)	169 (35)	1.6 (0.9–3.1)	*0.014*
IE, definite	2 (5)	41 (8)	0.6 (0.1–2.4)	0.56
IE, possible	22 (54)	107 (22)	4.1 (2.1–7.8)	*< 0.001*
IE, possible, treated as IE	1 (2)	6 (1)	2 (0.2–17)	0.44
Treatment, iv antibiotics (days)	6 (3–8)	6 (3–12)	n/a	0.60
Treatment, total (days)	12 (10–14)	14 (10–18)	n/a	0.15

The columns show a comparison between the MEfsB episodes without a previous EfsB but followed by a recurrent episode and episodes not followed by a new episode. Due to not being at risk of a recurrent EfsB, episodes, where the patient did not survive 30 days, were excluded, so were episodes with a previous EfsB infection. Differences in continuous variable were calculated with Wilcoxon’s rank sum test. In categorical variables, the differences were calculated with the chi-square test when applicable and Fisher’s exact test in other cases. Values are presented as proportions with percentage in parenthesis or medians with interquartile ranges in parenthesis. The odds ratios and their confidence intervals, in parenthesis, were calculated when applicable. A *p* value of < 0.05 was considered to be an indication of a significant difference. Significant differences in favor of the first of multiple episodes are shown in italics

### Is it possible to identify episodes preceding an IE episode?

MEfsBs, not diagnosed as IE during the first episode, followed by an episode diagnosed with IE, were compared to the rest of the episodes. The comparison was made after excluding episodes with IE, episodes in patients that did not survive 30 days, and all episodes where the patient had a history of a previous EfsB, 11 patients and 472 patients, respectively (Table 3 and S Fig. 1). The variables long symptom duration; predisposition for IE; heart murmur; growth in all, or the majority of, the BC bottles; and unknown origin of infection were significantly correlated with IE in univariable analysis. Positive NOVA score, DENOVA score, and DENOVA score with the cutoff ≥ 2 and possible IE were significantly more common. In 4 out of 11 of the patients (36%), a TEE had been performed and in 14% in the comparison group. No differences were seen in the treatment time (Table 3).

**Table 3:** Comparison of clinical characteristics and management in episodes followed by an IE episode compared to episodes without a following IE episode using univariable analysis

Characteristics	Episodes followed by IE (*n* = 11)	Episodes not followed by IE (*n* = 472)	Odds ratio (95% CI)	*p* value
Acquisition				
Community-acquired	4 (36)	108 (23)	1.9 (0.6–6.7)	0.29
Healthcare-associated	7 (64)	222 (47)	2.0 (0.6–6.8)	0.28
Nosocomial	0 (0)	142 (30)	n/a	0.04
Duration of symptoms ≥ 7 days	4 (36)	39 (8)	6.3 (1.8–23)	*0.011*
CIED	0 (0)	51 (11)	n/a	0.62
Predisposition	6 (55)	77 (16)	6.2 (1.8–21)	*0.005*
Predisposing cardiac condition	5 (45)	72 (15)	4.6 (1.4–16)	*0.019*
Prosthetic valve	3 (27)	40 (9)	4.0 (1.03–16)	0.065
Native valve disease	3 (27)	43 (9)	3.7 (0.96–15)	0.08
Previous IE	2 (8)	8 (2)	13 (2.4–69)	*0.019*
Intravenous drug user	2 (8)	5 (1)	20 (3.5–121)	*0.009*
Heart murmur	5 (45)	57 (12)	6.0 (1.8–21)	*0.007*
Fever ≥ 38°	8 (73)	398 (84)	0.50 (0.1–1.9)	0.39
Embolization	0 (0)	2 (0.4)	n/a	1.0
Number of positive cultures ≥ 2	9 (82)	86 (18)	20 (4.3–95)	*< 0.001*
Unknown origin of infection	8 (73)	129 (27)	7.1 (1.9–27)	*0.003*
Origin of infection	3 (27)	334 (71)	0.15 (0.04–0.6)	0.004
Urinary tract	2 (18)	216 (46)	0.26 (0.06–1.2)	0.07
CRI	1 (9)	22 (5)	2.0 (0.3–17)	0.42
DENOVA positive score	6 (55)	66 (14)	7.4 (2.2–25)	*0.002*
DENOVA cutoff ≥ 2	9 (82)	147 (31)	10 (2.1–47)	*0.001*
NOVA positive score	11 (100)	311 (66)	n/a	*0.019*
Management				
TTE performed	7 (64)	116 (25)	5.4 (1.5–19)	*0.008*
TEE performed	4 (36)	65 (14)	3.6 (1.02–13)	0.058
TTE or TEE performed	8 (73)	138 (29)	6.5 (1.7–25)	*0.004*
IE, possible	8 (73)	119 (25)	7.9 (2.1–30)	*< 0.001*
IE, possible, treated as IE	1 (9)	6 (1)	7.8 (0.9–71)	0.15
Treatment, iv antibiotics, (days)	7 (4–9)	5 (3–10)	n/a	0.60
Treatment, total (days)	12 (7–15)	13 (10–16)	n/a	0.61

Calculations were made after exclusion of all IE, all patients diseased within 30 days of positive BC, and episodes preceded by an EfsB. Differences in continuous variable were calculated with Wilcoxon’s rank sum test. In categorical variables, the differences were calculated with the chi-square test when applicable and Fisher’s exact test in other cases. Values are presented as proportions with percentage, in parenthesis, or medians with interquartile ranges, in parenthesis. The odds ratios and their confidence intervals, in parenthesis, were calculated when applicable. A *p* value of < 0.05 was considered to be an indication of a significant difference. Significant differences in favor of the first episode are shown in italics

All episodes later diagnosed with IE had a positive NOVA score, while the DENOVA score with the cutoff ≥ 2 identified 9/11 of the episodes, and with the prespecified cutoff ≥ 3, 6/11 were positive. The false positive results of the three scores were 311 (66%), 147 (31%), and 66 (14%), respectively (Table 3).

### Patients diagnosed with IE and with a following episode

Four patients with IE had a following Efs episode. The treatment times were 28 days, 30 days, 42 days, and 69 days of intravenous (iv) treatment. All were diagnosed with IE also during the following episode. The times to the next episodes were 66 days, 71 days, 302 days, and 350 days (data not shown and S Fig. 2).

### Microbiology

Efs isolates from patients with more than one episode of bacteremia were available from 31 patients (S Table 2 and S Fig. 1). Only frozen isolates that were viable for culture and where the coupled isolate from another episode was also available were sequenced. Among these 31 patients, 26 had two episodes and five patients had three episodes. In 28 of 31 patients, isolates of identical ST were found during all the episodes, three patients with three episodes and 25 with two episodes. One of these, patient 73 (S Table 2), had two nearly identical isolates in the two episodes, with only a minor difference in the *pst*S gene allele, a single locus variant, resulting in an ST change from ST 16 in the first episode to ST 1349 in the second episode.

The patients with non-similar ST between the episodes were one patient with two episodes with UTI; one iv drug user with an Efs IE during the first episode and, 350 days later, diagnosed with a second IE but with Efs of a different ST and *Streptococcus mitis* in BCs; and one patient with three episodes, with an isolate with one ST during the first episode and with two isolates of the same ST during the second and third episodes (S Table 2).

IE was found in more than one episode in isolates with STs 6, 21, 81, and 179. Isolates with STs 16, 26, 53, 55, 918, and 1357 were found in one IE episode each (Table 4).

**Table 4:** Episodes of MEfsB sorted according to ST and IE

Sequence type (ST)	Definite Duke IE		Total
	No	Yes	
6	7	3	10
16	2	1	3
19	2	0	2
21	2	2	4
26	0	1	1
30	2	0	2
40	3	0	3
53	1	1	2
55	2	1	3
64	4	0	4
81	1	2	3
179	5	3	8
191	2	0	2
203	1	0	1
273	1	0	1
295	2	0	2
330	1	0	1
875	2	0	2
918	1	1	2
1349	1	0	1
1355	2	0	2
1357	0	1	1
1358	2	0	2
Total	46	16	62

Episodes with PEfsB are not shown (see Table 1). Two or more episodes in a patient appear in columns and rows according to ST and IE or non-IE in each episode

## Discussion

The first of three main findings of this study was that 35% of the patients with a previous EfsB and presenting with a MEfsB were diagnosed with IE. In such patients, the clinician should persist in searching for an IE with TEE and possibly also other modalities [11].

Secondly, in univariable analysis, patients with episodes of MEfsB, not diagnosed as IE, but followed by a new episode of MEfsB, were found to be present with the same associated clinical characteristics as the IE risk factors described in patients diagnosed with IE during the same episode: long duration of symptoms; embolization; growth in all or the majority of the BCs; unknown origin of infection; predisposing heart condition, including prosthetic valve or native valve disease; and heart murmur [7]. However, all these patients are not suspected to have IE but could be oversimplified and be described to belong to two main categories. One group had a UTI during the first episode and a relapse in a new UTI during the second episode. The other group had an episode with unknown origin of infection, characteristics of an IE, without a TEE or was subjected to a TEE that was negative for IE, treated with a short course of antibiotics, and within 6 months, contracted a new episode diagnosed with IE or a new episode with unknown origin. Despite the known risk factors for IE, registered during clinical practice, TEE was not performed in many of these patients. The low proportion was probably due to the decentralized decision making and inappropriate routines concerning patients with EfsB, observations supported by two recent narrative reviews [17, 21]. An implementation of a routine to subject patients to TEE according to a risk factor analysis might decrease the risk of recurrence.

In the DENOVA study, we advocated for the use of the score to reach high sensitivity and specificity for IE and a low number needed to screen [7]. The NOVA score suggests TEE to be made in all EfsB episodes with multiple positive BCs or having an unknown focus of the infection [5] while Dahl et al. [6, 10, 17] suggest TEE to be made in all EfsB episodes, both polymicrobial and monomicrobial, due partly to the very high risk seen in the subgroup of community acquisition. In the present study, DENOVA has an insufficient sensitivity (55%) to be used during the first episode, if the aim is to identify all patients later diagnosed with IE. NOVA has a 100% sensitivity at the cost of a reduced specificity (31%) in the studied cohort. If the DENOVA cutoff would be lowered to ≥ 2, it would give a reasonable trade-off with 82% sensitivity and 69% specificity possibly useful in settings where TEE is not a limited resource. However, the sensitivity of TEE to detect a putative IE in these early suspected cases is not self-evident and has to be tested prospectively. The results of this study do not support any of the evaluation strategies, but we suggest at least a positive DENOVA score to be used in all MEfsBs, to decide if TEE should be performed. Independent of the strategy to do TEE, vigilance is called for from the patient and the clinician, after an episode of MEfsB, to react to any symptom compatible with a relapse of EfsB or IE within at least 6 months.

In the patients subjected to TEE but without findings typical to IE and a later recurrence with IE, no further evaluation with PET-CT or cardiac CT was done. We cannot tell whether these patient episodes represent missed IE due to insufficient sensitivity of the TEE, but it could be the case in some of the episodes. The European guidelines suggest search for cardiac changes and embolization with PET-CT and cardiac CT in cases of suspected IE but with a negative TEE [11] to increase the sensitivity to diagnose IE. We hypothesize that the implementation of that work-up has the potential to further reduce the number of relapses by diagnosing and treating some cases of IE.

The third main finding that the WGS, and the ST derived from it, shows that a vast majority of the patients with more than one episode of EfsB had identical isolates in consecutive episodes irrespective of whether the patient had IE or not at recurrence, thus implying a true relapse of the first infection. A relapse could have been caused by a previous insufficiently treated infection, in our study typically a UTI or an infection with unknown origin. Our original hypothesis that identical isolates of EfsB would indicate an IE in the second episode turned out to be true, but this was true also for the patients with other diagnoses, like UTI.

Other reflections that can be made from our results are that a MEfsB episode with a preceding PEfsB episode was likely to represent an IE (6/7 episodes). Furthermore, our study showed recurrent infections in 4 out of 69 episodes (6%) of IE, similar to the results of a contemporary study of recurrent IE [15] from Spain.

This is, to our knowledge, the largest study on multiple episodes of EfsB [10, 18] and the largest and the first to firmly establish the identity of the ST of multiple bacteremia isolates. Our principal observation from the clinical part of the study was that EfsB not diagnosed as IE often was diagnosed as IE during the relapse. This has not been described before. However, it is in line with the observation in Dahl et al. [10], where 6 patients in the non-IE group, with a negative echocardiography, had embolic events, indicating IE, and 13 (5%) had a new EfsB, a possible relapse with IE, not further described. This level of recurrences was similar to the findings in our study (6%).

The present study has several limitations. First, these analyses have been done post hoc. The collected cohort was originally assembled to develop support for making the decision whether TEE should be performed or not [7] and not to explore the hypotheses addressed in this study. Furthermore, the data on the associations between the clinical variables and the outcomes with recurrent infection or IE in the study did not permit a multivariable analysis and can thus not be claimed to be independently associated. The data only allowed univariable analysis. Finally, the routines in the clinical laboratories did not support the analysis of all coupled isolates with WGS and, possibly, there was a bias in which isolates were stored in the laboratories. However, we believe that some of the observations can be used in clinical practice to improve the diagnostic work-up in situations where a high risk of IE is identified.

In conclusion, we believe that our study can shed some light on the complicated clinical situations of EfsB and encourage to perform a rational evaluation of risk factors, like the DENOVA score, of patients in the cohort of EfsB. Finally, it can be emphasized that a recurrent infection with EfsB is often a true relapse and indicates IE.

## Supplementary Information

ESM 1

## Data Availability

*The datasets generated during and/or analyzed during the current study are not publicly available due to the conditions of the ethical approval but are available from the corresponding author on reasonable request.*
