# Supplementary material for: Recurrent bacteremia with Enterococcus faecalis, the clinical findings predicting endocarditis, and genomic characterization of the isolates: a retrospective cohort study
Source: Eur J Clin Microbiol Infect Dis. 2023 Jul 8;42(8):1001–9. doi: 10.1007/s10096-023-04636-3 (PMC10344971; doi:10.1007/s10096-023-04636-3)

Supplementary material

*Detailed description of the patients with a combination of poly- and monomicrobial infectious episodes*

Two episodes of monomicrobial EfsB episodes were followed by PEfs episodes. One patient had an initial wound infection and a vascular graft infection, followed by a graft infection during the PEfs episode (Table 1, row 5). The other patient was an iv-drug user with a tricuspid valve IE followed by another IE with growth of both Efs and *Steptococcus mitis* in BCs. Seven episodes were preceded by a PEfsB; in two of these the patient had a second episode of monomicrobial EfsB (Table 1, rows 2 and 3). The origin of the preceding PEfsB was abdominal or unknown. Full structured data were not available for the PEfsB, due to not being included in the cohort of MEfsB in this study.

Supplement tables

**Supplementary table 1:** Characteristics of the cohort and comparison of the episodes following an EfsB to other episodes using univariable analysis.

| **Characteristics** | **All (n=666)** | **Recurrent episodes (n=57)** | **Other**  **episodes**  **(n=609)** | **Odds ratio**  **(95% CI)** | ***P*-value** |
| --- | --- | --- | --- | --- | --- |
| Age (years) | 73 (65-81) | 76 (65-82) | 73 (65-81) | n/a | 0.53 |
| Sex (female) | 187 (28) | 12 (21) | 175 (29) | 0.7 (0.3-1.3) | 0.22 |
| Charlson score | 3 (1-5) | 2 (1-4) | 3 (1-5) | n/a | 0.93 |
| Acquisition |  |  |  |  |  |
| Community-acquired | 160 (24) | 8 (14) | 152 (25) | 0.5 (0.2-1.06) | 0.065 |
| Healthcare-associated | 326 (49) | 42 (74) | 284 (47) | 3.2 (1.7-5.9) | **<0.001** |
| Nosocomial | 180 (27) | 7 (12) | 173 (28) | 0.4 (0.2-0.8) | 0.009 |
| Duration of symptoms ≥7 days | 111 (17) | 24 (42) | 87 (14) | 4.4 (2.5-7.7) | **<0.001** |
| CIED | 72 (11) | 5 (9) | 67 (11) | 0.8 (0.3-2.0) | 0.60 |
| Predisposition | 155 (23) | 25 (44) | 130 (21) | 2.9 (1.6-5.0) | **<0.001** |
| Predisposing cardiac condition | 143 (22) | 24 (42) | 119 (20) | 3.0 (1.7-5.3) | **<0.001** |
| Prosthetic valve | 80 (12) | 15 (26) | 65 (11) | 3.0 (1.6-5.7) | **0.001** |
| Native valve disease | 82 (12) | 12 (21) | 70 (11) | 2.1 (1.04-4.1) | **0.035** |
| Previous IE | 26 (4) | 6 (11) | 20 (3) | 3.5 (1.3-9.0) | **0.018** |
| Intravenous drug user | 17 (3) | 2 (4) | 15 (2) | 1.4 (0.4-6.5) | 0.65 |
| Heart murmur | 131 (20) | 28 (49) | 103 (17) | 4.7 (2.7-8.3) | **<0.001** |
| Fever ≥38 degrees | 535 (80) | 40 (70) | 495 (81) | 0.5 (0.3-0.99) | 0.044 |
| Embolization | 12 (2) | 4 (7) | 8 (1) | 5.7 (1.7-19) | **0.012** |
| Number of positive cultures ≥2 | 191 (29) | 35 (61) | 156 (26) | 4.6 (2.6-8.1) | **<0.001** |
| Unknown origin of infection | 248 (37) | 37 (65) | 211 (35) | 3.5 (2.0-6.2) | **<0.001** |
| Origin of infection | 402 (60) | 16 (28) | 386 (63) | 0.22 (0.1-0.4) | <0.001 |
| Gastrointestinal | 91 (14) | 1 (2) | 90 (15) | 0.1 (0.01-0.8) | 0.006 |
| Urinary tract | 253 (38) | 14 (25) | 239 (39) | 0.5 (0.3-0.94) | 0.029 |
| CRI | 24 (4) | 0 (0) | 24 (4) | n/a | 0.25 |
| Wound | 21 (3) | 1 (2) | 20 (3) | 0.5 (0.1-4) | 1.0 |
| Other foci | 18 (3) | 4 (7) | 14 (2) | 3.2 (1.02-10) | 0.60 |
| Joint infection | 4 (1) | 1 (2) | 3 (0.5) | 3.6 (0.4-35) | 0.30 |
| Spondylodiscitis | 12 (2) | 3 (5) | 9 (1) | 3.7 (0.97-14) | 0.08 |
| DENOVA positive score | 165 (25) | 33 (58) | 132 (22) | 5.0 (2.8-8.7) | **<0.001** |
| Management |  |  |  |  |  |
| TTE performed | 208 (31) | 32 (56) | 176 (29) | 3.1 (1.8-5.5) | **<0.001** |
| TEE performed | 142 (22) | 27 (47) | 115 (19) | 3.9 (2.2-6.9) | **<0.001** |
| TTE or TEE performed | 240 (36) | 35 (61) | 205 (34) | 3.1 (1.8-5.5) | **<0.001** |
| IE, definite diagnosis | 69 (10) | 20 (35) | 49 (8) | 6.2 (3.3-11) | **<0.001** |
| IE, possible | 168 (25) | 18 (32) | 150 (25) | 1.4 (0.8-2.5) | 0.25 |
| IE, possible, treated as IE | 10 (2) | 2 (4) | 8 (1) | 2.7 (0.6-13) | 0.21 |
| Treatment, iv antibiotics (days) | 6 (3-12) | 11 (5-28) | 6 (3-11) | n/a | **<0.001** |
| Treatment, total (days) | 13 (10-17) | 15 (13-32) | 13 (9-17) | n/a | **<0.001** |

Footnote: The first column shows the entire cohort. The second and third columns show a comparison between the episodes with a preceding episode of EfsB and all other episodes. Differences in continuous variable were calculated with Wilcoxon’s rank sum test. In categorical variables, the differences were calculated with the χ^₂^-test when applicable and Fisher’s exact test in other cases. Values are presented as proportions with percentage, in parenthesis, or medians with interquartile ranges, in parenthesis. The odds ratios and their confidence intervals, in parenthesis, were calculated when applicable. A *p-*value of <0.05 was considered to be an indication of a significant difference. Significant differences in favor of the recurrent episodes are shown in bold.

**Supplementary table 2**. Each episode of monomicrobial EfsB is shown together with its corresponding last episode. Further, the ST of the isolates of the episodes and the most significant diagnoses are all shown on each row.

| **Preceding episode** | **Sequence type ST** | **UTI** | **Unknown focus** | **IE** | **Last episode** | **Sequence type ST** | **UTI** | **Unknown focus** | **IE** |  |
| --- | --- | --- | --- | --- | --- | --- | --- | --- | --- | --- |
| Pat 43:1 | 0 | 0 | 1 | 0 | Pat 43:2 | 0 | 0 | 1 | 0 |  |
| Pat 73:1 | 16 | 0 | 1 | 0 | Pat 73:2 | 1349 | 1 | 0 | 0 |  |
| Pat 76:1 | 179 | 0 | 1 | 0 | Pat 76:2 | 179 | 0 | 0 | 1 |  |
| Pat 94:1 | 64 | 0 | 1 | 0 | Pat 94:2 | 64 | 0 | 1 | 0 |  |
| Pat 105:1 | 0 | 0 | 1 | 0 | Pat 105:2 | 0 | 1 | 0 | 0 |  |
| Pat 108:1 | 0 | 1 | 0 | 0 | Pat 108:2 | 0 | 1 | 0 | 0 |  |
| Pat 125 | 64 | 0 | 0 | 1 | Pat 125:p | 64 | 0 | 0 | 1 |  |
| Pat 155:1 | 1358 | 0 | 1 | 0 | Pat 155:2 | 1358 | 0 | 1 | 0 |  |
| Pat 224:1 | 0 | 0 | 1 | 0 | Pat 224:2 | 0 | 0 | 1 | 0 |  |
| Pat 227:1 | 53 | 0 | 1 | 0 | Pat 227:2 | 53 | 0 | 0 | 1 |  |
| Pat 267:1 | 1355 | 1 | 0 | 0 | Pat 267:2 | 1355 | 0 | 1 | 0 |  |
| Pat 287:1 | 21 | 0 | 1 | 0 | Pat 287:2 | 21 | 0 | 1 | 0 |  |
| **Pat 299** | **1357** | **0** | **0** | **1** | **Pat 299:p** | **63** | **0** | **0** | **1** |  |
| Pat 309:1 | 6 | 1 | 0 | 0 | Pat 309:2 | 6 | 1 | 0 | 0 |  |
| Pat 362:1 | 179 | 0 | 1 | 0 | Pat 362:2 | 179 | 0 | 1 | 0*** | * |
| Pat 382:1 | 19 | 1 | 0 | 0 | Pat 382:2 | 19 | 1 | 0 | 0 |  |
| Pat 389:1 | 0 | 1 | 0 | 0 | Pat 389:2 | 0 | 1 | 0 | 0 |  |
| Pat 390:1 | 0 | 0 | 1 | 0 | Pat 390:2 | 0 | 0 | 0 | 1 |  |
| **Pat 393:1** | **16** | **1** | **0** | **0** | **Pat 393:2** | **273** | **1** | **0** | **0** | ***** |
| Pat 401:1 | 0 | 1 | 0 | 0 | Pat 401:2 | 0 | 0 | 1 | 0 |  |
| Pat 413:1 | 0 | 0 | 1 | 0 | Pat 413:2 | 0 | 0 | 1 | 0 |  |
| Pat 421:1 | 0 | 0 | 0 | 0 | Pat 421:2 | 0 | 0 | 0 | 0 |  |
| Pat 424:1 | 6 | 0 | 1 | 0 | Pat 424:2 | 6 | 0 | 0 | 1 |  |
| Pat 468:1 | 0 | 1 | 0 | 0 | Pat 468:2 | 0 | 1 | 0 | 0 |  |
| **Pat 493:1** | **40** | **1** | **0** | **0** | **Pat 493:3** | **81** | **0** | 0 | **1** | ****** |
| Pat 493:2 | 81 | 0 | 0 | 0 | Pat 493:3 | 81 | 0 | 0 | 1 |  |
| Pat 507:1 | 30 | 1 | 0 | 0 | Pat 507:2 | 30 | 1 | 0 | 0 | * |
| Pat 511:p | 26 | 0 | 0 | 0 | Pat 511 | 26 | 0 | 0 | 1 |  |
| Pat 512:1 | 0 | 0 | 0 | 0 | Pat 512:3 | 0 | 1 | 0 | 0 |  |
| Pat 512:2 | 0 | 1 | 0 | 0 | Pat 512:3 | 0 | 1 | 0 | 0 |  |
| Pat 519:p | 81 | 0 | 0 | 0 | Pat 519 | 81 | 0 | 0 | 1 |  |
| Pat 539:1 | 0 | 1 | 0 | 0 | Pat 539:2 | 0 | 1 | 0 | 0 |  |
| Pat 551:1 | 0 | 0 | 1 | 0 | Pat 551:2 | 0 | 0 | 0 | 1 |  |
| Pat 560:1 | 875 | 1 | 0 | 0 | Pat 560:2 | 875 | 0 | 1 | 0 |  |
| Pat 585:p | 0 | 0 | 0 | 0 | Pat 585 | 0 | 0 | 0 | 1 |  |
| Pat 588:1 | 0 | 0 | 1 | 0 | Pat 588:2 | 0 | 0 | 1 | 0 |  |
| Pat 613:p | 918 | 0 | 0 | 0 | Pat 613:2 | 918 | 0 | 0 | 1 |  |
| Pat 613:1 | 918 | 0 | 1 | 0 | Pat 613:2 | 918 | 0 | 0 | 1 |  |
| 202 | 0 | 0 | 1 | 0 | 204 | 0 | 0 | 1 | 0 |  |
| 271 | 191 | 0 | 1 | 0 | 275 | 191 | 0 | 1 | 0 |  |
| 296 | 6 | 1 | 0 | 0 | 297 | 6 | 0 | 1 | 0 |  |
| 308 | 295 | 1 | 0 | 0 | 310 | 295 | 1 | 0 | 0 |  |
| 322 | 55 | 0 | 1 | 0 | 325 | 55 | 0 | 0 | 1 |  |
| 323 | 55 | 0 | 1 | 0 | 325 | 55 | 0 | 0 | 1 |  |
| 331 | 6 | 1 | 0 | 0 | 333 | 6 | 0 | 0 | 1 |  |
| 376 | 16 | 0 | 0 | 1 | 380 | 0 | 0 | 0 | 1 |  |
| 434 | 179 | 0 | 1 | 0 | 439 | 179 | 0 | 0 | 1 |  |
| 489:p | 6 | 0 | 0 | 0 | 492 | 6 | 0 | 0 | 1 |  |
| 490 | 6 | 0 | 1 | 0 | 492 | 6 | 0 | 0 | 1 |  |
| 560 | 21 | 0 | 0 | 1 | 561 | 21 | 0 | 0 | 1 |  |
| 654 | 0 | 0 | 0 | 0 | 662 | 0 | 0 | 0 | 1 |  |
| 659 | 0 | 0 | 0 | 1 | 662 | 0 | 0 | 0 | 1 |  |
| 677 | 40 | 0 | 1 | 0 | 679 | 40 | 0 | 1 | 0 |  |
| 789:p | 64 | 0 | 0 | 0 | 792 | 64 | 0 | 0 | 0*** |  |
| 827 | 179 | 0 | 1 | 0 | 830 | 179 | 0 | 0 | 1 |  |
| 891:p | 0 | 0 | 0 | 0 | 891 | 0 | 0 | 0 | 1 |  |

Foot note:

*lacking the isolate from the third episode

**isolate from the first episode is unique, second and third identical

***septic arthritis or spondylodiscitis in last episode

Isolates from different episodes that were not identical are shown in bold face. Identical numbers in two following “Last episode” column rows refer to the same episode preceded by two episodes in column “Preceding episode”.

Supplement figures

**Supplement figure 1:** Flowchart describing the comparisons of the cohort in different tables. Groups in red boxes were compared in the table. See also the text in the Result section for exclusions.

Entire cohort S table 1 Table 2 Table 3

Table 4 and S table 2

**Supplement figure 2**. Time between episodes


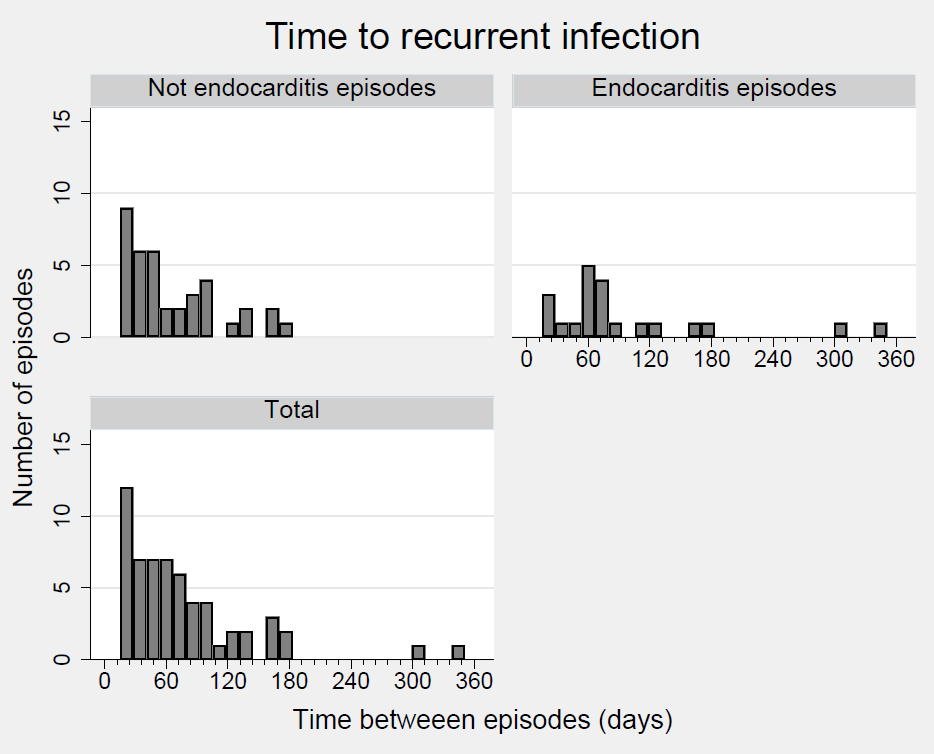

Supplement: Supplementary file 1 — ESM 1 [file 10096_2023_4636_MOESM1_ESM.docx]
